# Iontophoresis effects of two-step self-etch and total-etch systems on dentin permeability and sealing of composite restoration under simulated pulpal pressure

**DOI:** 10.1186/s12903-022-02632-1

**Published:** 2022-12-08

**Authors:** Orapin Ajcharanukul, Peeraya Santikulluk, Palat Sasingha, Sirithorn Sabpawat, Kanokporn Sukyanan

**Affiliations:** 1 Department of Stomatology, Faculty of Dentistry, Srinakharinwirot University, Asokemontri Road, Wattana, Bangkok, 10110 Thailand; 2 The Dean Office, Faculty of Dentistry, Srinakharinwirot University, Bangkok, Thailand

**Keywords:** Dental bonding system, Dentin permeability, Iontophoresis, Resin composite, Hybrid layer

## Abstract

**Background:**

Studies demonstrated the bond strength enhancement and the decrease in degradation of the adhesive interface after applying either self-etch adhesives or two-step, etch-and-rinse adhesives under an electric field. However, the presence of dentinal fluid driven by the pulpal pressure in vivo is a profounding factor affecting both the sealing ability and bond strength of adhesives. This study aimed to evaluate the effect of three-step etch-and-rinse and two-step self-etch adhesives when applied with iontophoresis under simulated pulpal pressure on the permeability of dentin, resin infiltration, and the sealing ability of resin composite.

**Methods:**

The experiments were done on 32 recently extracted premolars, randomly assigned into four groups (n = 8) according to two adhesive systems (SBMP and SE), applied following the manufacturer’s instructions (control) for two groups or with iontophoresis for the others (SBMPi and SEi). For the iontophoresis, the anodal current was applied at 75 μA for 20 s through the cavity electrode during the bond. The fluid flow rate of dentin was recorded after cavity preparation (smear-layer-covered dentin; T1), bonding (T2), and composite restoration (T3) during the maintained pulpal pressure of 20 mm Hg. The flow rates were expressed as a percentage relative to the initial smear-layer-covered value for each specimen. Results were analyzed using repeated measures ANOVA. Scanning electron microscopy (SEM) was performed to observe the resin/dentin interface.

**Results:**

There were no significant increases in the mean flow rates from T1 to T3 in the SBMP (P = 0.355), while these changes in the SE were significant between T1 (100%) and T2 (166.77%) and T1 and T3 (221.16%) (P = 0.002; one-way RM ANOVA; Holm-Sidak test). For the iontophoresis groups, the mean flow rates decreased significantly from T1 to T2 and T1 to T3 of both SBMPi (T2 = 86.43, and T3 = 79.53; P < 0.001) and SEi groups (T2 = 87.96, and T3 = 81.48; P = 0.004). The iontophoresis of both adhesives produced the optimal resin infiltration with improved quality of the hybrid layer and resin tags.

**Conclusions:**

SBMP bonded with or without iontophoresis performed better sealing ability than SE under the same condition. Both adhesives applied with anodal iontophoresis significantly decreased the dentin permeability, contributing to the improved resin infiltration.

## Background

Under physiologic pulpal pressure causing a continuous outward fluid flow through the dentin, studies demonstrated that restorative procedures, including dentin bonding and composite restoration, could not perfectly seal the tooth structure [1, 2]. When the hydraulic conductance technique was used to determine the adhesive permeability, various systems of dental adhesives allowed fluid transudation differently across the bonded dentin [3]. The results revealed that a three-step, etch-and-rinse adhesive such as Scotchbond Multi-Purpose (SBMP) sealed dentin better than other adhesives, whereas, for the self-etch adhesive, the appropriate dentin sealing could be achieved using Clearfil SE Bond (SE). However, most adhesives do not seal dentin as well as that smear layers [3, 4]. They permeate the demineralized dentin structure mainly via a diffusion mechanism which is highly variable depending on the adhesive system used, the dentin substrate, and the intervention of the operator [5, 6]. These factors result in different degrees of the poor seal within the hybrid layer along the adhesive interface, increasing bond degradation, and subsequent loss of retention of the composite restoration [7]. Some procedures are suggested to improve the impregnation of the resin monomers into dentin, such as using an additional layer of hydrophobic resin [8] and applying multiple layers of adhesives [9]. Although these were proven beneficial for some bonding agents [10], the iontophoretic effects on the bonding application could provide another option for further studies of the selected bonding agents to be used under certain clinical situations.

The use of iontophoresis to enhance drug delivery through the tooth structure was widely investigated, with promising results indicating the possibilities to apply to the field of restorative dentistry [11–13]. Studies demonstrated the bond strength enhancement and the decrease in degradation of the adhesive interface after applying either self-etch adhesives [14–16] or two-step, etch-and-rinse adhesives [17–20] under an electric field [21]. The intensity of the applied current contributing to the significantly higher bond strength was from 0 to 110 μA. Since the three-step adhesive system was considered effective in reducing dentin permeability [3], the use of iontophoresis to enhance its penetration into dentin may increase the sealing ability and be a reference condition. Furthermore, the presence of dentinal fluid driven by the pulpal pressure in vivo is a substantial factor affecting both the sealing ability [1, 3] and bond strength of adhesives [22, 23]. Recent evidence indicated that the rate and direction of continuous outward fluid flow through dentin could be changed when a direct current (DC) was passed from the dentin surface into the pulp using different polarities and intensities of the current [24]. Such phenomenon, so-called electroosmosis, is produced during the iontophoresis through a tissue causing an enhanced delivery of agents through dentin. Also, a DC device is necessary to guarantee the constant current applied during the drug delivery of the iontophoresis because the compositions of the tooth structure are highly variable among individuals, resulting in differences in the electrical resistances of the tooth areas [14, 21, 25]. Therefore, the bonding agent delivered with a DC iontophoresis can ensure its effect on the dentinal fluid movement with increasing infiltration of the adhesive interface.

By determining the dye penetration qualitatively through the resin-dentin interface under simulated pulpal pressure, N Gharizadeh, A Kaviani and S Nik [26] demonstrated that the microleakage scores reduced significantly after applying etch-and-rinse adhesive (Single Bond) with the electric current of 15 μA. However, details of such effects on the other adhesive types, instrument set, application time, electrode polarity, and the quantitative evaluation of the dentin fluid flow under the experimental condition were absent, prompting the challenge in the potential application. Therefore, this study aimed to quantitatively evaluate the effect of three-step etch-and-rinse and two-step self-etch adhesives when applied with iontophoresis under simulated pulpal pressure on the permeability of dentin, resin infiltration, and the sealing ability of resin composite. The null hypothesis was that the application of iontophoresis with bonding agents created comparable effects to that of the conventional methods on the sealing ability of dentin under simulated pulpal pressure.

## Methods

The experimental protocol was exempted from permission by the ethical committee of Srinakharinwirot University (SWUEC-321/2562X). The required sample size for ANOVA at the different conditions was determined based on previous experiments [27] by using the SigmaPlot 11.0 (Systat Software Inc., San Jose, CA, USA). The minimum size was calculated as 5–7 for each treatment group with a desired power of 0.8 and a significance level of 0.05. The experiments were done on 32 extracted human premolars recently extracted for orthodontic purposes. Teeth were free of caries or restorations and stored in 0.1% thymol solution at 4 °C for up to 4 weeks before use.

### Sample preparation and fluid flow measurement

**Fig. 1:**
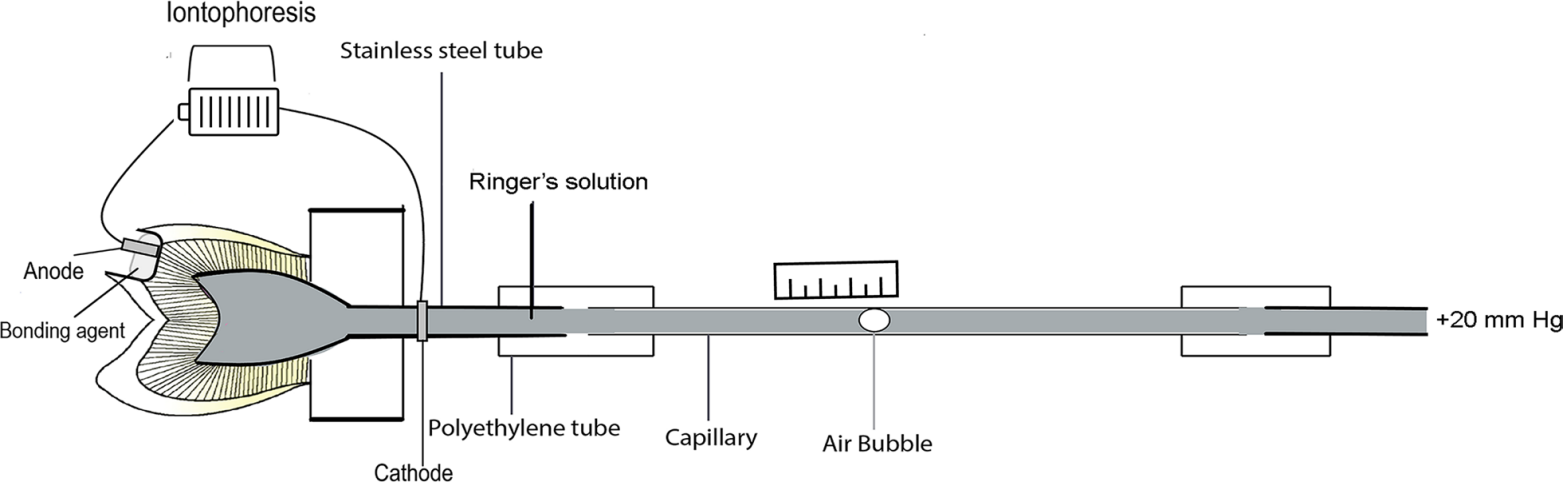
Diagram of the experimental setup to record fluid flow rates during which the bonding agents were applied. A magnifying lamp was used to measure the moving fluid between the pulpal cavity and dentine under each of the experimental conditions by recording the movement of a small air bubble introduced into the capillary. The fluid flow rate was calculated from a distance moved by the bubble in time (mm/min)

The sample preparation and testing were carried out by two operators while the other recorded fluid flow rates. The tooth was cut transversely 2 mm below the cemento-enamel junction with a diamond disc under streaming water. The coronal pulpal tissue was removed with fine tweezers, then irrigated with water using a triple syringe to remove any remaining tissue. Dentine was exposed at the tip of the buccal cusp by cutting a cavity (diameter 4 mm, depth 3 mm) with an air-rotor and straight cylinder diamond burs (Nos. 201 and 204, Intensive1, Viganello-Lugano, Switzerland) under cooling water. A fluid flow measurement was set up in each specimen as shown in Fig. 1. The crown was sealed with cyanoacrylate cement (Alteco Inc., Osaka, Japan) to an acrylic block into which had been sealed a stainless-steel tube (18 G, o.d. 1.27 mm, i.d. 0.84 mm). Each tooth crown and acrylic block were held together in a vertical direction until the glue was set to prevent excess in the pulp chamber. The pulp chamber, stainless steel tube, and capillary were filled with Ringer’s solution and kept in the same horizontal plane during each flow rate measurement. The set-up was connected to a manometer and maintained at a pressure of 20 mm Hg above atmospheric to represent the normal tissue fluid pressure of the pulp in vivo [28]. Prior to measurements of fluid flow, the cavity floor was manually polished using a small piece of 600-grit abrasive paper and water for 30 s to obtain a standardized smear layer [19, 20].

Samples were randomly assigned into four groups (n = 8) according to the adhesive systems, (1) SBMP (3 M ESPE, St. Paul, MN, USA) and (2) SE (Kuraray, Osaka, Japan) (Table 1) applied following the manufacturers instruction for the control groups, and the use of iontophoresis during the bond application, (3) SBMPi, and (4) SEi groups. Each adhesive was light-cured for 10 s with a LED light-curing unit at 1200 mW/cm^2^ (D-2000, APOZA; New Taipei City, Taiwan). Estelite Quick (Tokuyama, Tokyo, Japan) was used as a composite restoration for the prepared cavity (Table 1). Each increment of 1.5–2 mm was light-cured for 20 s using the same light-curing unit. For the SBMPi and SEi groups, an iontophoresis machine was used to pass a D.C. current of 75 μA for 20 s between the cavity electrode and the stainless-steel needle (return electrode) inserted into the cannula beyond the glass capillary (Fig. 1). The machine measured the electrical resistance along with the passing current at the end of the current application.

**Table 1:** Materials used in the study

Material	Classification	Composition	Application
Adper Scotchbond Multi-purpose (SBMP; 3 M ESPE, St. Paul, MN, USA)	Three-step total-etch adhesive	*Etchant* 35% phosphoric acid *Primer* HEMA, polyalkenoic acid polymer, water *Bond* HEMA, Bis-GMA, tertiary amines, photoinitiator	Acid etch for 15 s, water rinse for 10 s, gently air dry for 5 s Apply primer for 10 s, gently air dry for 5 s Apply bond. Gently air dry Light cure 10 s
Clearfil SE Bond (SE; Kuraray, Osaka, Japan)	Two-step self-etch adhesive	*Primer* 10-Methcryloyloxydecyl Dihydrogen phosphate (MDP), HEMA, hydrophilic dimethacrylates, photoinitiator, amine, water *Bond* MDP, Bis-GMA, HEMA, hydrophobic dimethacrylate, colloidal silica, photoinitiator	Apply primer for 20 s, gently air dry for 5 s Apply bond, Gentle air dry Light cure 10 s
Estelite quick (Tokuyama, Tokyo, Japan)	Nano-filled composite resin	Bis-GMA, TEGDMA, silica zirconia fillers, silica-titania fillers, photoinitiators	Resin composite placement through incremental technique with each 2.0 mm increment light-cured

*HEMA* (hydroxyethyl)methacrylate, *BisGMA* bisphenol A glycidylmethacrylate, *MDP* 10-methacryloyloxydecyl dihydrogen phosphate, *TEGDMA* triethylene glycol dimethacrylate

The fluid flow, the movement of a small air bubble introduced into the capillary, was recorded by using a microscope and graticule during the continuously maintained pulpal pressure of 20 mm Hg (Fig. 1). The flow rates were obtained from each sample after the cavity preparation (smear layer-covered dentin; T1), after adhesive (T2), and after composite restoration (T3). The flow direction that occurred during anodal iontophoresis with each bonding was observed to determine if the electroosmosis could affect the motion of the air bubble in the glass capillary. To compensate for the high variations in adhesive permeability, dentin structure, and composition among the samples, the flow measurements for each sample were also expressed as a percentage increase or decrease relative to the initial smear-layer-covered value. Thus, each sample served as its control.

### Scanning electron microscopy (SEM) preparation

After finishing the fluid flow measurements, each sample was longitudinally fractured to examine the nature of the resin/tooth interface under the SEM. Representative samples of each group (n = 2) were sectioned into two halves using a low-speed diamond disc under saline irrigation to clarify the distribution of the resin tags. The sectioned surfaces were polished with 1500 and 3000-grit silicon carbide sandpaper for 30 s before submitting to ultrasound water cleaning for 1 min. Subsequently, they were etched with 35% phosphoric acid gel for 1 min, followed by deproteinization in 5.25% NaOCl for 30 min as modified from N Nakabayashi and DH Pashley [29]. All SEM samples were dehydrated in ascending grades of ethanol (50%, 75%, 95%, and 100% for 10 min each) and subjected to a critical-point dryer for 30 min. Then, they were coated with Au/Pt in a sputter-coater and examined under the SEM (JEOL, Model 5400, Tokyo, Japan).

The representative SEM images of all samples were also used to measure the remaining dentine thickness (RDT) between the floor of the cavity and the closest point of the pulp chamber along the dentinal tubules using measurement features of the SEM imaging software.

### Statistical analysis

The data of the flow rates and the percentages increase or decrease relative to the initial smear layer covered dentin values (T1) were summarized as mean ± 1 S.D. The data obtained from each group were analyzed statistically using one-way, repeated measures and analysis of variance (one-way RM ANOVA). Where this showed a significant effect, the Holm-Sidak test was used for pairwise comparisons between the individual means. Pearson correlations between percentage fluid conductance changes after adhesive and composite restoration of each group were determined by using linear regression analyses. P values < 0.05 were considered significant.

## Results

**Fig. 2:**
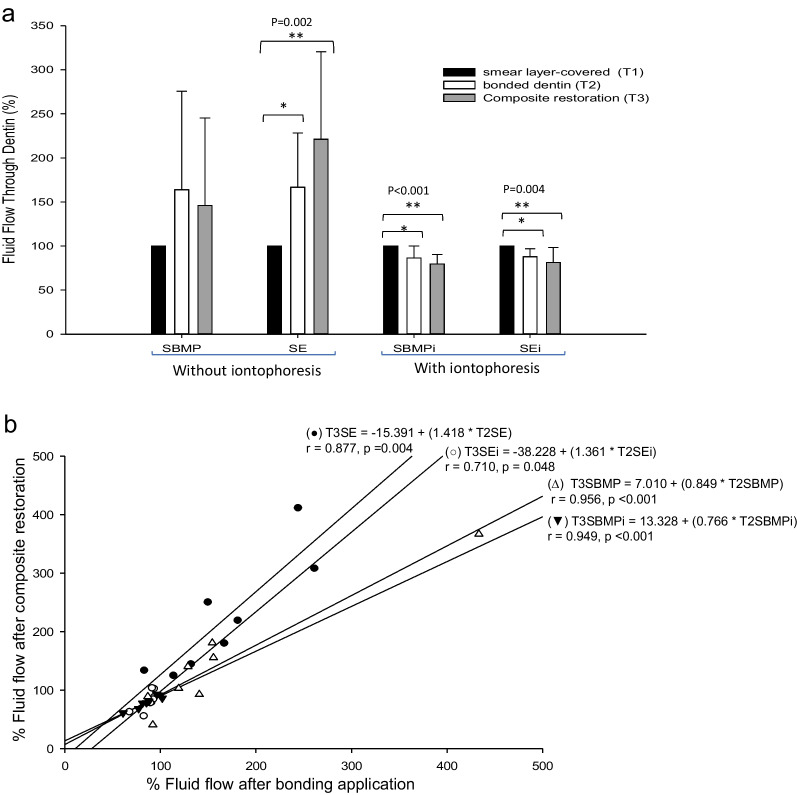
**a** Bar charts showing the means and standard deviations of the percentage change of fluid flow rate from baseline obtained from that in the smear layer-covered dentin recorded with different adhesives and application either with or without iontophoresis. **b** Linear regression analyses comparing the percentage changes of fluid flow after bond associated with that obtained after composite restoration. SE = Clearfil SE Bond; SBMP = Scotchbond Multi-Purpose; SEi = Clearfil SE Bond applied with iontophoresis; SBMPi = Scotchbond Multi-Purpose applied with iontophoresis. *P* < 0.05 indicates statistical significance

The mean flow rates recorded at T1, T2, and T3 of each group are shown in Table 2. The fluid conductance expressed as percentage changes of the baseline smear-layer-covered values for each group were summarized in Fig. 2a. In the treatment groups without iontophoresis, there were no significant increases from T1 to T3 in the SBMP (P = 0.355), while these changes of the SE group were significant between T1 (100%) and T2 (166.77%) and T1 and T3 (221.16%) (P = 0.002; one-way RM ANOVA; Holm-Sidak test). This revealed that the conventional application of SBMP could seal the cavity’s wall as effectively as that smear layer-covered condition, whereas the sample bonded with the SE resulted in a significant increase in permeability even after composite restoration. For the iontophoresis, the mean percentage changes of the flow rates decreased significantly from T1 to T2 and T1 to T3 of both SBMPi (T2 = 86.43, and T3 = 79.53; P < 0.001) and SEi groups (T2 = 87.96, and T3 = 81.48; P = 0.004) (Fig. 2a), indicating that the application of anodal iontophoresis during the bonding step of both adhesives improved dentin sealing after adhesive and after composite restoration significantly better than that in the baseline smear layer.

**Table 2:** Effect of iontophoresis applied with bonding agents on fluid flow rate across smear layer-covered dentin (T1), bonded dentin (T2) and after composite restoration (T3)

Dentin condition	Conventional application (mm/min)		Iontophoresis application (mm/min)	
	SE	SBMP	SEi	SBMPi
T1	1.67 (1.3)^A^	3.58 (2.58)^a^	1.27 (0.43)^A^	1.28 (0.63)^a^
T2	2.54 (1.85)^B^	4.15 (2.68)^a^	1.11 (0.39)^AB^	1.0 (0.65)^ab^
T3	3.06 (1.8)^B^	3.68 (2.68)^a^	1.03 (0.43)^B^	0.94 (0.65)^b^

*SE* Clearfil SE Bond; *SBMP* Scotchbond Multi-Purpose; *SEi* Clearfil SE Bond applied with iontophoresis; *SBMPi* Scotchbond Multi-Purpose applied with iontophoresis

In the same column, subgroups with different letter superscripts indicate a significant difference (p < 0.05)

When the percentage fluid conductance at T2 was plotted against T3 of each group, significant positive correlations were found and expressed as linear regression analyses in Fig. 2b. Furthermore, observations of the bubble movement in the capillary during the application of adhesives demonstrated that it was paused when the current was applied at the bonding in all samples of the iontophoresis groups, while this was outwardly continued during the adhesive application without iontophoresis.

The means ± SDs of the remaining dentine thicknesses obtained from all groups were not statistically significant differences (SE, 1.27 ± 0.37 mm; SEi, 1.2 ± 0.3 mm; SBMP, 1.1 ± 0.38 mm; SBMPi, 1.87 ± 0.34 mm; P > 0.05; one-way RM ANOVA).

**Fig. 3:**
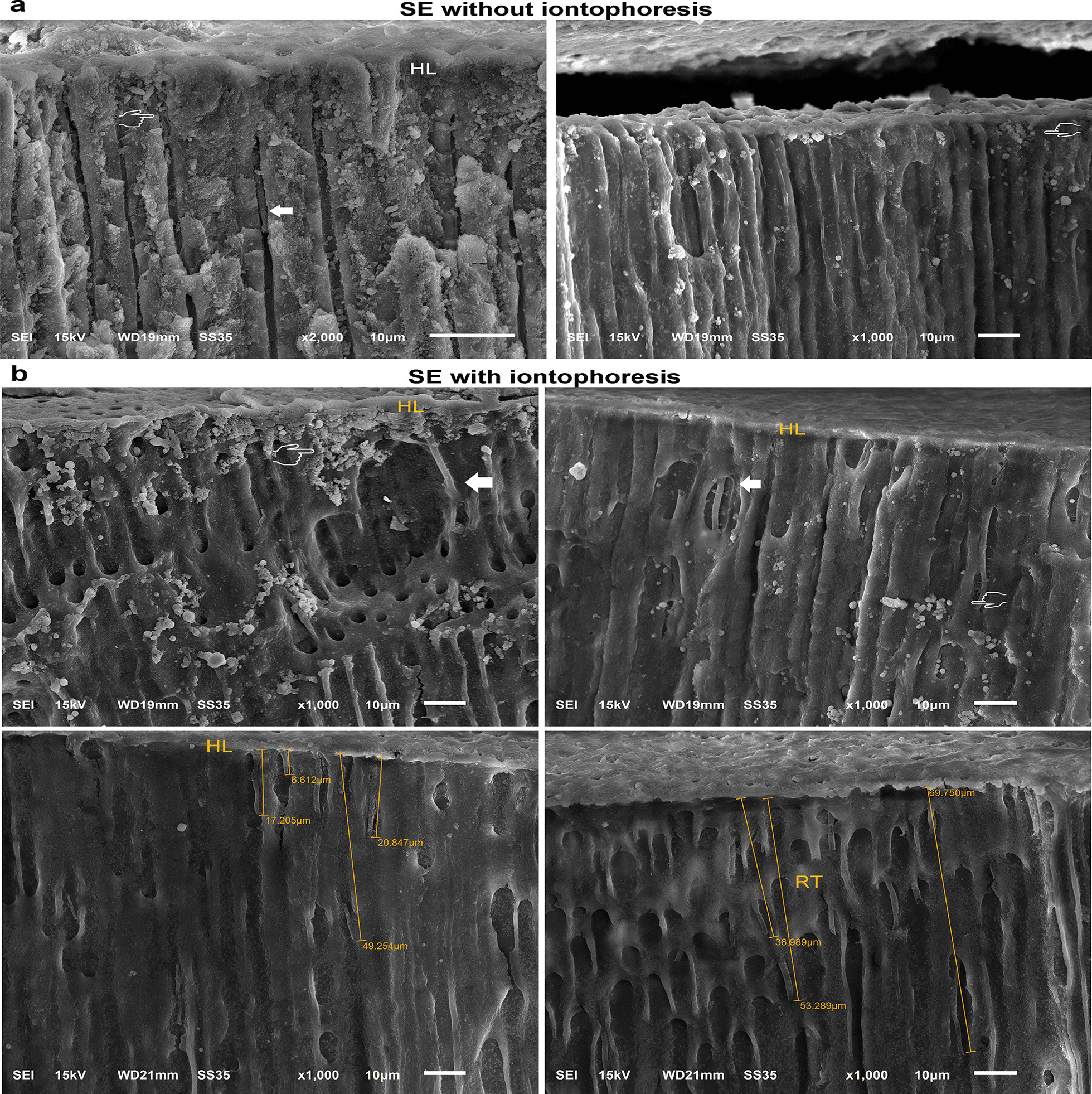
Representative SEM micrographs of resin-dentin bonds with SE applied without **a** and with iontophoresis **b** obtained from the fractured samples. Smear plugs (pointers) and tags (white arrow) are less marked in (**a**). When the iontophoresis was used to apply the SE, the thick and continuous hybrid layer was observed along with tags up to 70 µm long (**b**). HL = hybrid layer; RT = resin tags

**Fig. 4:**
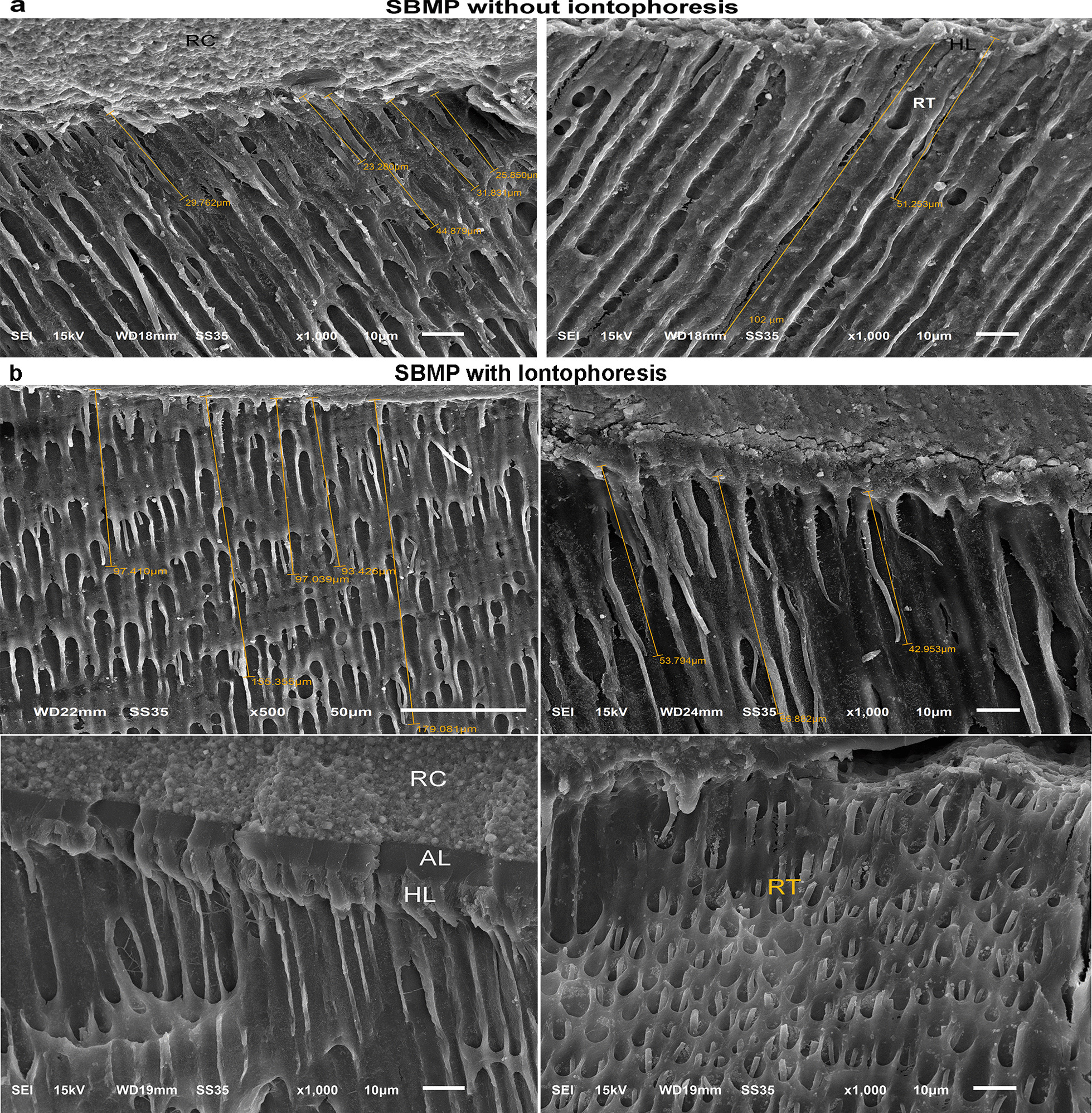
Representative SEM micrographs showing the resin-dentin bonds with SBMP applied without **a** and with iontophoresis (**b**). The resin tags were clearly detectable with a thin and irregular hybrid layer varying in thickness, approximately between 1 and 4 µm (**a**). When the SBMP was bonded with iontophoresis, the higher density and longer tags with a conical swelling at the base are clearly visible (**b**). RC = resin composite; AL = adhesive layer

**Fig. 5:**
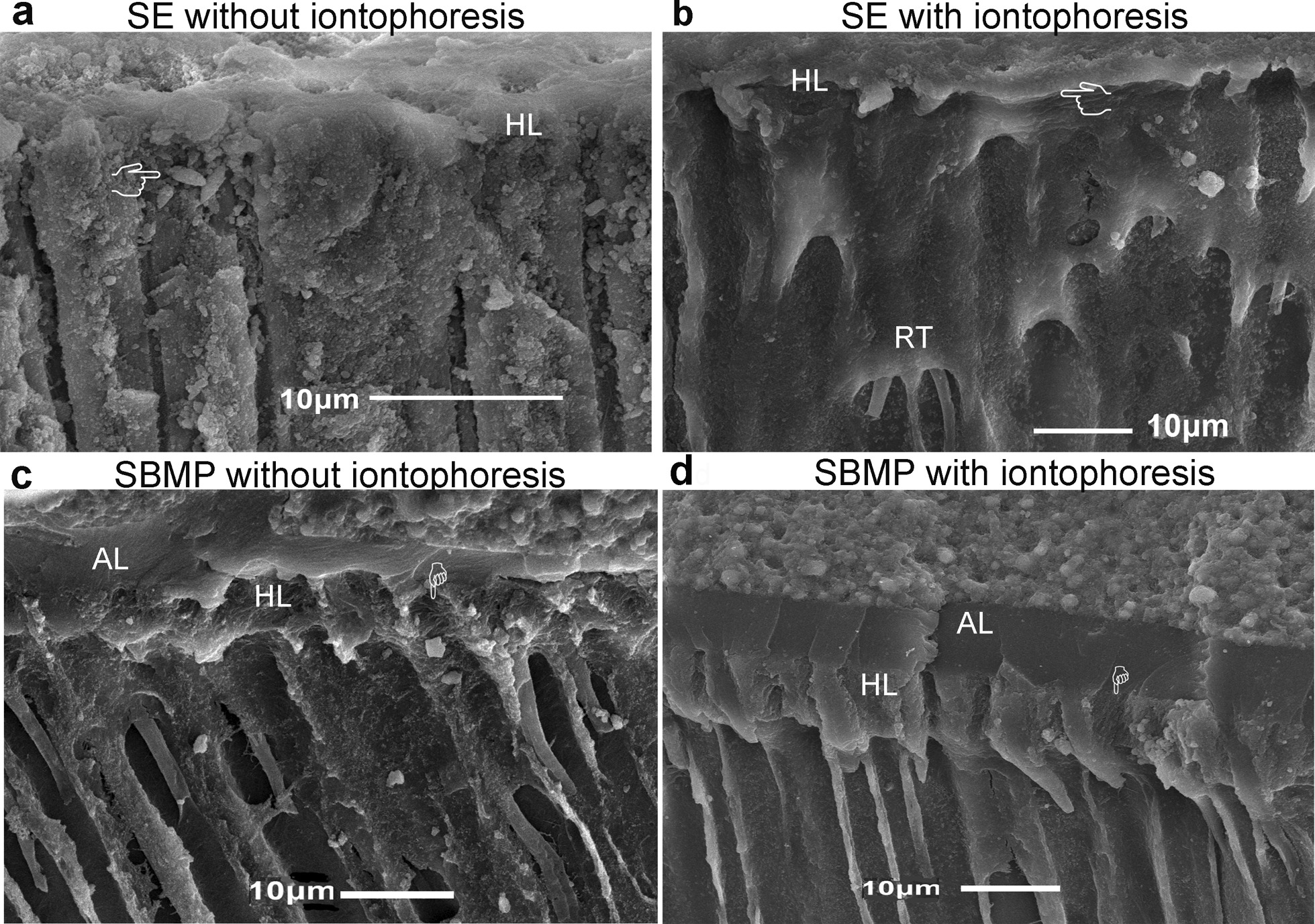
Representative resin-dentin interfaces of SE **a**, **b** and SBMP **c**, **d** were applied without and with iontophoresis. The increased thickness and perfectly infiltrated HL (pointers) are evident in both adhesives applied with iontophoresis **b**, **d** when compared with those bonded with conventional methods (**a**, **c**). More homogenous resin infiltration of demineralized collagen fibrils and smear plugs were present in the HL (**b**). The overall AL and HL integrities were improved with iontophoresis (**d**)

Representative SEM photomicrographs of bonded interfaces are shown in Fig. 3, 4, 5, and 6. Figure 3 is representative of resin-dentin bonds with SE applied without (Fig. 3a) and with iontophoresis (Fig. 3b). SE adhesives partially infiltrate no further than the smear layer, revealing a regional difference in morphology of the hybrid layer (HL) and the rare evidence of resin tags (Figs. 3a and 5a). The bonded interface obtained with SE applied with iontophoresis has more condensed HL and tags up to 70 µm long with an increased accumulation of smear plugs inside the dentinal tubules (Figs. 3b and 5b). Resin tags are clearly detectable in the SBMP sample with partially infiltrated HL (Figs. 4a and 5c). For the SBMPi samples, the well-infiltrated HL (approximately 8 µm in thickness), including the longer and greater numbers of tags up to 179 µm, have been observed. These tags typically show distinct conical enlargement at their bases (Figs. 4b and 5d), suggesting that SBMP applied with iontophoresis could fully envelop the deepest portion of the demineralized collagen fibrils within the HL.

**Fig. 6:**
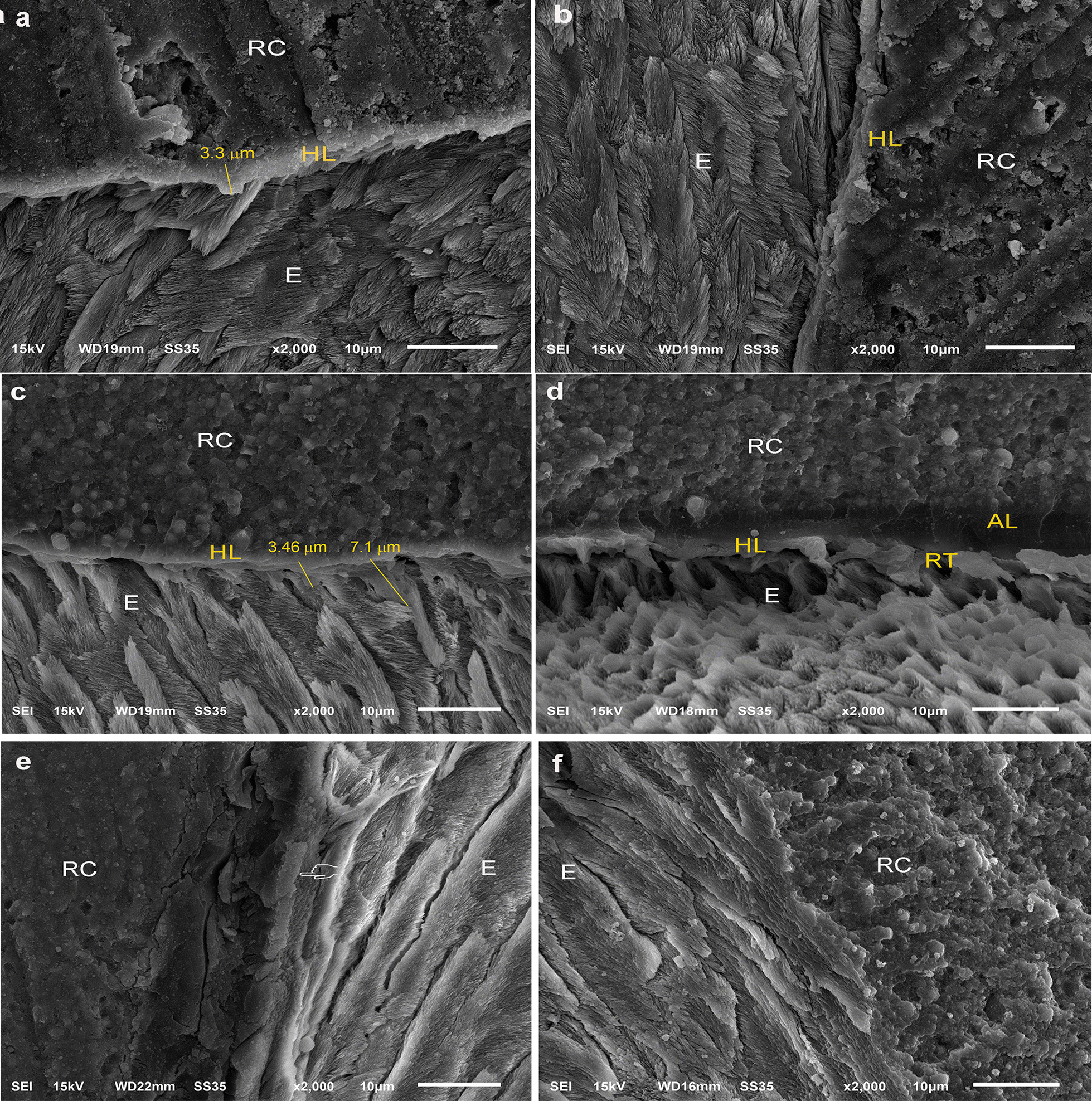
Representative resin-enamel interfaces of SBMP applied without **a**, **b** and with **c**–**f** iontophoresis respectively. The size and number of resin tags are more evident in the SBMP samples applied with iontophoresis (**c**–**f**). E = enamel

The resin-enamel interfaces of SBMP samples are more detectable than those in SE. The representative SEM images of the SBMP applied without and with iontophoresis were demonstrated in Fig. 6. There are regional differences in the presence of the HL regardless of the SBMP’s application mode. However, the increases in size and number of resin tags, including the HL thickness, are more obvious in the SBMPi (Fig. 6c–f) than those in the SBMP samples (Fig. 6a, b).

## Discussion

Under simulated pulpal pressure, the application of either three-step etch-and-rinse (SBMP) or two-step self-etch (SE) adhesives using the anodal iontophoresis significantly decreased the fluid flow after bond and composite restoration when compared with those applied according to the manufacturer’s instructions without the electric current. The results well corresponded with the SEM analyses showing the optimal resin infiltration with a longer and greater number of resin tags obtained after applying electric current during the bond application of both adhesives. Considering the significant linear relationship between the permeabilities attained after bond and composite restoration, the slopes of the regression equations obtained after SBMP and SBMPi were lower than those in SE and SEi samples (Fig. 2b), demonstrating a better sealing ability after composite restoration when using SBMP as adhesive. Furthermore, a slight decrease of the slopes found in both types of adhesives applied with iontophoresis indicates that the improved seal obtained after using the anodal current in the bonding step of both agents also facilitated the immediate reduction of the tooth permeability after composite restoration (Fig. 2b).

Although study designs, electric currents, and types of adhesives were different, this study was consistent with the previous results showing significant improvements in the bond strength and the bonding quality after applying self-etch [14–16] or two-step, etch-and-rinse adhesives [17–20] with the electric currents varying from 0 to 110 μA [21]. The electric-current-assisted application of etch-and-rinse adhesive (Single Bond) also decreased microleakage scores of the class V cavities under simulated pulpal pressure [26], which corresponded to the results of this study. However, the present data could evidently demonstrate the degree of flow rate changes following the crucial steps of restorative procedures, the applications of two different adhesive systems, and composite restoration under pulpal pressure. Such findings were well associated with the possible differences between the demineralization front and the adhesive interface, as revealed by the SEM images. Studies show that the pulpal pressure in teeth in vivo caused increased permeability and decreased bond strength of the tooth-restoration bond [22, 23]. When the bonding of both adhesives with the anodal current of 75 μA for 20 s was performed in the current study, the outward flow created during the applied pressure was paused, indicating the decrease in outward dentinal fluid movement into the dentinal tubules and therefore reducing its penetration into polymerized hydrophilic monomer. Therefore, the electroosmotic flow resulting from the applied current could enhance the resin infiltration by decelerating the outward flow driven by the simulating pulpal pressure of 20 mm Hg across the dentin. This finding entirely agrees with the recent study showing that the direction of the electroosmotic flow with both polarities of applied current was from anode to cathode and that electroosmosis can deliver both uncharged and charged molecules through the dentin to the pulp [13, 24]. Furthermore, in a pilot study, it was found that the anodal current at the minimum of 250 μA caused the inward flow from dentin into the pulp during the bond application, which this also consistent with the previous finding that the fluid flow rates through dentin produced by electroosmosis increased as the current intensity was increased regardless of the test solution’s composition [24]. Since the higher current intensity (≥ 250 μA) causing inward fluid flow could raise the possibility of the cytotoxicity of adhesives in vivo [30], the applied current in this study was set at approximately 75 μA.

The outward flow rates obtained after bonding and restoration in the SE group were significantly increased, while these changes were not different in the SBMP samples, indicating the less ability of SE to prevent dentin fluid from permeating across the polymerized adhesive. The finding was in accord with the previous results also measuring the dentinal fluid flow during composite restoration [2, 31] and that by Carrilho et al. [32], showing the better sealing ability of the smear layer than adhesive resins. The possible explanations involved the presence of porosities with the low integrity of the bonded smear layer-cover dentin (Fig. 3a), allowing the fluid permeation, also reported in the literature [1, 23]. The increased fluid conductance across the tooth-restoration interface in the SE group could be explained by the fact that the initially weak bond strength of the SE to the cavity wall probably caused the higher degree of polymerization shrinkage stress of resin composite [33]. Moreover, it could be the result of complex interactions of several stimuli generated during the restorative procedure, including the structure and composition of the tooth, such as thermal expansion from the light sources [31].

Using smear layer-covered dentin as a reference condition in this study demonstrated the increased sealing efficacy of the tooth-restoration bond using both SBMP and SE applied with anodal iontophoresis under pulpal pressure. Even though the conventional application of SBMP generally performed better laboratory results than SE [3, 34], the increase in immediate bonding effectiveness produced by two different adhesive systems employed with anodal iontophoresis in this study suggested the comparable efficacy between SBMP and SE. Thus, the use of simplified adhesive, SE, applied with iontophoresis is recommended to decrease the number of clinical steps with additional bonding effectiveness.

It appears that the prolonged application time was found to increase the immediate microtensile bond strength of two-step etch-and-rinse adhesive systems [35]. The limitation of this study was partly from the less application time of the bonding agents applied with the conventional methods compared to those with iontophoresis, requiring a duration of 20 s for conducting the electric current. In addition, the ultrastructure of the resin infiltration pattern should also be characterized using the transmission electron microscope [36, 37], as this would help confirm the bonding quality improved with iontophoresis. The fact that dentin is more complex, with high variables in the viscosities of dentinal fluid in vivo resulting in different degrees of wetter dentin than in vitro studies [38], could also affect the bonding efficacy for the clinical application. However, due to the simplicity of the clinical procedure and the safety of the minimum intensity of the electric current, in vivo use of anodal iontophoresis during bond application should be further investigated for possible use in dental practice.

## Conclusions

Under simulated pulpal pressure, the anodal iontophoresis could cause a brief pause of the continuous outward fluid flow through the dentin, enhancing the resin infiltration at the adhesive/dentin interface of both three-step (SBMP) and two-step (SE) adhesives applied to the dentin as revealed by the SEM images. The process then contributed to the substantial reduction of the dentin permeability compared with the conventional methods. SBMP and SE, when applied with iontophoresis, produced a similar increase in the degree of sealing ability compared to that in conventional procedure.

## Data Availability

The datasets used and/or analyzed during the current study are available from the corresponding author on reasonable request.

## Abbreviations

SBMP: Scotchbond Multi-Purpose
SE: Clearfil SE Bond
DC: Direct current
SBMPi: Scotchbond Multi-Purpose applied with iontophoresis
SEi: Clearfil SE Bond applied with iontophoresis
HEMA: (Hydroxyethyl)methacrylate
BisGMA: Bisphenol A glycidylmethacrylate
MDP: 10-Methacryloyloxydecyl dihydrogen phosphate
TEGDMA: Triethylene glycol dimethacrylate
SEM: Scanning electron microscopy
RDT: Remaining dentine thickness;
RMANOVA: Repeated measures, analysis of variance
T1: Smear layer-covered dentin
T2: After adhesive
T3: After composite restoration
HL: Hybrid layer
RT: Resin tags
RC: Resin composite
AL: Adhesive layer
E: Enamel

## References

[1] Banomyong D, Palamara JE, Messer HH, Burrow MF (2008). Sealing ability of occlusal resin composite restoration using four restorative procedures. Eur J Oral Sci.

[2] Kim SY, Ferracane J, Kim HY, Lee IB (2010). Real-time measurement of dentinal fluid flow during amalgam and composite restoration. J Dent.

[3] Nawareg MM, Zidan AZ, Zhou J, Chiba A, Tagami J, Pashley DH (2015). Adhesive sealing of dentin surfaces in vitro: a review. Am J Dent.

[4] Breschi L, Maravic T, Cunha SR, Comba A, Cadenaro M, Tjaderhane L, Pashley DH, Tay FR, Mazzoni A (2018). Dentin bonding systems: from dentin collagen structure to bond preservation and clinical applications. Dent Mater.

[5] Giachetti L, Scaminaci Russo D, Bertini F, Pierleoni F, Nieri M (2007). Effect of operator skill in relation to microleakage of total-etch and self-etch bonding systems. J Dent.

[6] Frankenberger R, Krämer N, Petschelt A (2000). Technique sensitivity of dentin bonding: effect of application mistakes on bond strength and marginal adaptation. Oper Dent.

[7] Breschi L, Mazzoni A, Ruggeri A, Cadenaro M, Di Lenarda R, De Stefano DE (2008). Dental adhesion review: aging and stability of the bonded interface. Dent Mater.

[8] Bahari M, Mohammadi N, Alizadeh Oskoee P, Savadi Oskoee S, Davoodi F (2017). Effect of an extra layer of hydrophobic resin on the microleakage of Cl V composite resin restorations with a universal adhesive system. J Investig Clin Dent.

[9] Yokoyama M, Takamizawa T, Tamura T, Namura Y, Tsujimoto A, Barkmeier WW, Latta MA, Miyazaki M (2021). Influence of different application methods on the bonding effectiveness of universal adhesives to dentin in the early phase. J Adhes Dent.

[10] Osorio R, Osorio E, Aguilera FS, Tay FR, Pinto A, Toledano M (2010). Influence of application parameters on bond strength of an “all in one” water-based self-etching primer/adhesive after 6 and 12 months of water aging. Odontology.

[11] Ikeda H, Suda H (2013). Facilitatory effect of AC-iontophoresis of lidocaine hydrochloride on the permeability of human enamel and dentine in extracted teeth. Arch Oral Biol.

[12] Puapichartdumrong P, Ikeda H, Suda H (2003). Facilitation of iontophoretic drug delivery through intact and caries-affected dentine. Int Endod J.

[13] Smitayothin TL, Vongsavan K, Rirattanapong P, Kraivaphan P, Vongsavan N, Matthews B (2015). The iontophoresis of lignocaine with epinephrine into carious dentine for pain control during cavity preparation in human molars. Arch Oral Biol.

[14] Breschi L, Mazzoni A, Pashley DH, Pasquantonio G, Ruggeri A, Suppa P, Mazzotti G, Di Lenarda R, Tay FR (2006). Electric-current-assisted application of self-etch adhesives to dentin. J Dent Res.

[15] Toledano M, Mazzoni A, Monticelli F, Breschi L, Osorio E, Osorio R (2011). ElectroBond application may improve wetting characteristics of etched dentine. J Dent.

[16] Chen H, Fu D, Yang H, Liu Y, Huang Y, Huang C (2014). Optimization of direct currents to enhance dentine bonding of simplified one-step adhesive. Eur J Oral Sci.

[17] Pasquantonio G, Tay FR, Mazzoni A, Suppa P, Ruggeri A, Falconi M, Di Lenarda R, Breschi L (2007). Electric device improves bonds of simplified etch-and-rinse adhesives. Dent Mater.

[18] Mazzoni A, Visintini E, Vita F, Pasquantonio G, Saboia VP, Ruggeri A, Di Lenarda R, Dorigo E, Breschi L (2009). ElectroBond improves immediate dentin microtensile bond strength of two etch-and-rinse adhesives. J Adhes Dent.

[19] Maciel CM, da Rosa Rinhel MF, Abuna GF, Pacheco RR, da Silva-Concílio LR, Baroudi K, Sinhoreti MAC, Vitti RP (2021). Resin composite adhesion to dentin using different curing lights and adhesive systems applied under electric current. Clin Oral Investig.

[20] Guarda MB, Di Nizo PT, Abuna GF, Catelan A, Sinhoreti MAC, Vitti RP (2020). Effect of electric current-assisted application of adhesives on their bond strength and quality. J Adhes Dent.

[21] Maciel CM, Souto TCV, Pinto BA, Silva-Concilio LR, Baroudi K, Vitti RP (2021). Adhesive systems applied to dentin substrate under electric current: systematic review. Restor Dent Endod.

[22] Rosales-Leal JI, de la Torre-Moreno FJ, Bravo M (2007). Effect of pulp pressure on the micropermeability and sealing ability of etch & rinse and self-etching adhesives. Oper Dent.

[23] Sauro S, Pashley DH, Montanari M, Chersoni S, Carvalho RM, Toledano M, Osorio R, Tay FR, Prati C (2007). Effect of simulated pulpal pressure on dentin permeability and adhesion of self-etch adhesives. Dent Mater.

[24] Kijsamanmith K, Vongsavan N, Matthews B (2020). Electroosmosis in human dentine in vitro. Arch Oral Biol.

[25] Chunhacheevachaloke E, Ajcharanukul O (2016). Effects of conducting media and gender on an electric pulp test. Int Endod J.

[26] Gharizadeh N, Kaviani A, Nik S (2010). Effect of using electric current during dentin bonding agent application on microleakage under simulated pulpal pressure condition. Dent Res J (Isfahan).

[27] Ajcharanukul O, Oranratmanee K, Thitikunakorn S (2010). Effect of different osmotic stimuli on fluid flow before and after self-etching adhesive application. J Adhes Dent.

[28] Tanapitchpong R, Chunhacheevachaloke E, Ajcharanukul O (2020). In vivo and in vitro study of enamel fluid flow in human premolars. Arch Oral Biol.

[29] Nakabayashi N, Pashley DH (1998). Hybridization of dental hard tissues.

[30] Ratanasathien S, Wataha JC, Hanks CT, Dennison JB (1995). Cytotoxic interactive effects of dentin bonding components on mouse fibroblasts. J Dent Res.

[31] Ratih DN, Palamara JE, Messer HH (2007). Dentinal fluid flow and cuspal displacement in response to resin composite restorative procedures. Dent Mater.

[32] Carrilho MR, Tay FR, Sword J, Donnelly AM, Agee KA, Nishitani Y, Sadek FT, Carvalho RM, Pashley DH (2007). Dentine sealing provided by smear layer/smear plugs vs. adhesive resins/resin tags. Eur J Oral Sci.

[33] Stansbury JW, Trujillo-Lemon M, Lu H, Ding X, Lin Y, Ge J (2005). Conversion-dependent shrinkage stress and strain in dental resins and composites. Dent Mater.

[34] De Munck J, Van Landuyt K, Peumans M, Poitevin A, Lambrechts P, Braem M, Van Meerbeek B (2005). A critical review of the durability of adhesion to tooth tissue: methods and results. J Dent Res.

[35] Reis A, de Carvalho CP, Vieira LCC, Baratieri LN, Grande RHM, Loguercio AD (2008). Effect of prolonged application times on the durability of resin–dentin bonds. Dent Mater.

[36] Hanabusa M, Mine A, Kuboki T, Momoi Y, Van Landuyt KL, Van Meerbeek B, De Munck J (2011). TEM interfacial characterization of an experimental self-adhesive filling material bonded to enamel/dentin. Dent Mater.

[37] Mine A, De Munck J, Van Ende A, Poitevin A, Matsumoto M, Yoshida Y, Kuboki T, Van Landuyt KL, Yatani H, Van Meerbeek B (2017). Limited interaction of a self-adhesive flowable composite with dentin/enamel characterized by TEM. Dent Mater.

[38] Chinajitphan N, Ajcharanukul O, Kijsamanmith K, Vongsavan N, Matthews B (2021). Time-course of the effect of potassium oxalate in the treatment of hypersensitive dentine in man. Arch Oral Biol.

